# Synthesis and Characterization of Novel Resveratrol Butyrate Esters That Have the Ability to Prevent Fat Accumulation in a Liver Cell Culture Model

**DOI:** 10.3390/molecules25184199

**Published:** 2020-09-14

**Authors:** You-Lin Tain, Li-Cheng Jheng, Sam K. C. Chang, Yu-Wei Chen, Li-Tung Huang, Jin-Xian Liao, Chih-Yao Hou

**Affiliations:** 1 Department of Pediatrics, Kaohsiung Chang Gung Memorial Hospital and Chang Gung University College of Medicine, Kaohsiung 833, Taiwan; tainyl@hotmail.com (Y.-L.T.); litung.huang@gmail.com (L.-T.H.); 2 Institute for Translational Research in Biomedicine, Kaohsiung Chang Gung Memorial Hospital and Chang Gung University College of Medicine, Kaohsiung 833, Taiwan; 3 Department of Chemical and Materials Engineering, National Kaohsiung University of Science and Technology, Kaohsiung 811, Taiwan; lcjheng@nkust.edu.tw; 4 Experimental Seafood Processing Laboratory, Costal Research and Extension Center, Mississippi State University, Pascagoula, MS 39567, USA; schang@fsnhp.msstate.edu; 5 Department of Food Science, Nutrition and Health Promotion, Mississippi State University, Starkville, MS 39762, USA; 6 Department of Medicine, Chang Gung University, Linkow 333, Taiwan; naosa720928@gmail.com; 7 Department of Seafood Science, National Kaohsiung University of Science and Technology, Kaohsiung 811, Taiwan; j0920181@gmail.com

**Keywords:** resveratrol butyrate ester, resveratrol, butyric acid, Steglich esterification, prevent fat accumulation

## Abstract

To facilitate broad applications and enhance bioactivity, resveratrol was esterified to resveratrol butyrate esters (RBE). Esterification with butyric acid was conducted by the Steglich esterification method at room temperature with *N*-ethyl-*N*′-(3-dimethylaminopropyl) carbodiimide (EDC) and 4-dimethyl aminopyridine (DMAP). Our experiments demonstrated the synthesis of RBE through EDC- and DMAP-facilitated esterification was successful and that the FTIR spectra of RBE revealed absorption (1751 cm^−1^) in the ester region. ^13^C-NMR spectrum of RBE showed a peak at 171 ppm corresponding to the ester group and peaks between 1700 and 1600 cm^−1^ in the FTIR spectra. RBE treatment (25 or 50 μM) decreased oleic acid-induced lipid accumulation in HepG2 cells. This effect was stronger than that of resveratrol and mediated through the downregulation of p-ACC and SREBP-2 expression. This is the first study demonstrating RBE could be synthesized by the Steglich method and that resulting RBE could inhibit lipid accumulation in HepG2 cells. These results suggest that RBE could potentially serve as functional food ingredients and supplements for health promotion.

## 1. Introduction

Resveratrol (RE) (trans-3,5,4′-trihydroxystilbene) is a phenolic stilbenoid compound with a C6–C2–C6 structure with three hydroxyl groups. It has been found in over 70 types of plants, including grape skin, grape seeds, giant knotweed, peanut, cassia seed, passion fruit, white tea, plums, and peanuts. It has powerful antioxidant activity and plays a role in the defense against pathogen infection, injury, and abiotic stress [1,2,3,4,5,6,7]. RE has been used in medicines, dietary supplements, and functional foods, and the excellent health benefits of RE have also been confirmed by several studies. It has preventive effects on oxidative stress, inflammation, and cardiovascular disease, and shows anti-carcinogenic activity [3,8,9,10]. Furthermore, RE has been proven to reduce the risk of developing diseases by inhibiting advanced glycation end product formation [11].

Based on its beneficial health effects, several studies focused on the use of RE in medicines, dietary supplements, and functional foods [12]. However, some studies have failed to confirm these beneficial effects, possibly due to RE’s high absorption but low bioavailability in vivo [8,13,14], which limits its development for therapeutic applications. Therefore, research is needed to improve the bioavailability of RE. The effects of RE depend on the microenvironment; the presence of copper ions can promote DNA damage and have anti-tumor effects [15]. To improve its biological activity, RE has been esterified using 12 different fatty acids, and derivatives of varying chain lengths and degrees of unsaturation were produced (C3:0–C22:6). RE esters with long-chain fatty acids (C18:0 and C18:1) show higher antioxidant activity in the DPPH radical scavenging assay [16], whereas those with short-chain fatty acids (SCFAs; C3:0, C4:0, and C6:0) show higher antioxidant activity in the ABTS radical cation scavenging assay. Therefore, esterification of RE may improve its functional performance, and the effect of esterification depends on the position and number of esterification substitutions. However, the effect of esterification on the bioactivity of RE is still unclear and requires further research. Furthermore, SCFAs have recently become popular targets of scientific research aiming to link gut microbiota to pathological conditions and potential health-beneficial effects in humans [17,18]. SCFAs are primarily produced through carbohydrate fermentation and also through protein and amino acid decomposition. The majority of SCFAs (95%) are represented by acetic acid (C2), propionic acid (C3), and butyric acid (C4) [19]. There is increasing evidence that SCFAs play an important role in effective absorption from the colonic lumen, and they represent 10% of the human daily energy intake [20]. Unlike acetic and propionic acid, which are mainly absorbed into the blood stream, butyric acid serves as a principal energy source for colonocytes, and its derivatives have many applications in chemical, food, pharmaceutical, perfume, and animal feed industries. Therefore, supplementation with SCFAs such as butyrate may increase microbiota metabolism [20,21].

RE is limited due to its low bioavailability in vivo, and it has been demonstrated that esterification may increase bioactivity [22,23]. Therefore, it is imperative to develop fast and simple esterification methods to synthesize RE butyrate esters. Traditional esterification methods, such as Fischer esterification, require the treatment of a carboxylic acid with an alcohol in the presence of a dehydrating agent. The equilibrium constant for such reactions is approximately five for typical esters, for example, ethyl acetate. Sulfuric acid is a typical catalyst for this otherwise slow reaction. Many other acids are also used as catalysts such as polymeric sulfonic acids. Since esterification is highly reversible, the yield of the ester can be improved using alcohol in large excess, using a dehydrating agent, or by removing the water by physical means. This method is useful in specialized organic synthetic operations but is considered too hazardous and expensive for large-scale applications. Furthermore, because of the limitations in the preparation of large amounts of resveratrol ester derivatives using conventional plant extraction procedures, chemical synthesis is one of the best methods for large-scale production. Therefore, Steglich esterification, which is a method of forming esters under mild conditions, may be a good method. This method is popular in peptide synthesis, where the substrates are sensitive to harsh conditions such as high heat. In this study, RE esters made with butyrate were produced using Steglich esterification techniques. The chemical structure of resveratrol butyrate esters (RBE) after esterification was characterized by mass spectrometry (MS), Fourier-transform infrared spectroscopy (FTIR), and nuclear magnetic resonance (NMR) analysis. In addition, the mechanism by which RBE inhibits fat accumulation was also evaluated using the HepG2 cell model, which might be interesting for potential health promotion and disease risk reduction.

## 2. Results and Discussion

**Figure 1:**
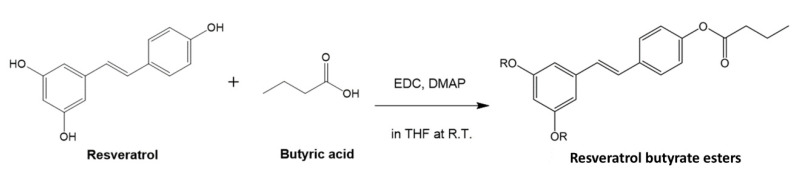
Synthesis of resveratrol butyrate esters. EDC, (1-ethyl-3-(3-dimethylaminopropyl) carbodiimide; DMAP, 4-N, *N*-dimethyl amino pyridine; THF, tetrahydrofuran; R.T., room temperature.

As shown in Figure 1, resveratrol butyrate esters (RBE) were synthesized from RE and butyl acid by Steglich esterification using *N*-ethyl-*N*′-(3-dimethylaminopropyl) carbodiimide (EDC) and 4-dimethylaminopyridine (DMAP). Compared to the traditional esterification reactions, this ester coupling reaction that is carried out at room temperature (about 28–30 °C) may prevent RE deactivation or degradation. *N*,*N*′–dicyclohexylcarbodiimide (DCC) is one of the most widely used activating and dehydrating agents in Steglich esterification, which was developed in 1978 by Neises and Steglich [24]. However, in preliminary experiments, we found that it was difficult to completely remove DCC from the resulting RE esters using either gravity filtration or gel column chromatography methods because during the coupling reaction, DCC dicyclohexylurea (DCU) is formed as a byproduct, which is insoluble in most organic solvents [25] but not soluble in water. Therefore, after esterification, it was difficult to separate RE and DCU from water. It has been reported that water-soluble EDC, an alternative to DCC, can be used for the esterification of phenol derivatives [26] and can be removed after esterification because it is water-soluble [25]. Hence, we replaced DCC with EDC for the esterification in the present study. Because EDC, DMAP, and the urea byproduct can be dissolved in water, we found that they did not significantly contaminate our RE butyrate esters after a simple purification by precipitating the product from deionized water.

**Figure 2:**
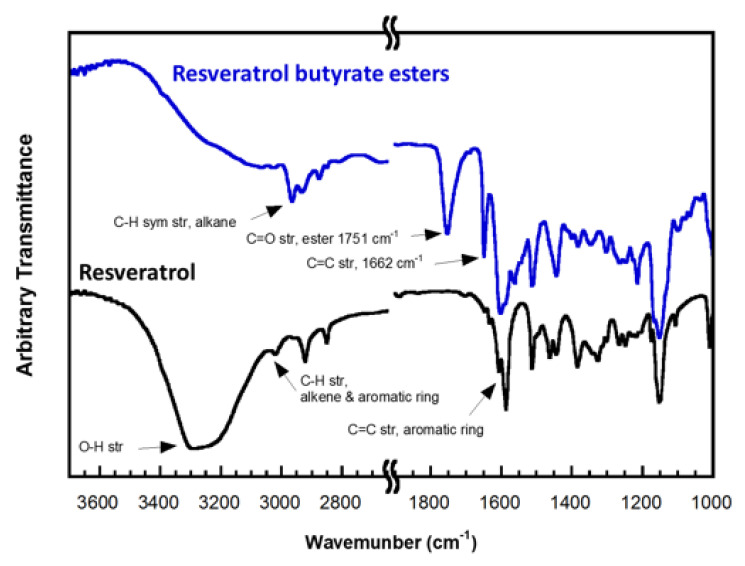
FTIR spectra of resveratrol and resveratrol butyrate esters.

The Fourier-transform infrared spectroscopy (FTIR) spectra of RE and RBE are compared in Figure 2. The FTIR spectra of RE matches that reported by Porto I. et al. [27]. We found that a broad band in the region of 3400–3200 cm^−1^, assigned to the O–H stretching, disappeared and a new absorption band at 1751 cm^−1^, ascribed to the C=O stretching of the ester, appeared in the spectrum of RBE. This comparative finding proved that the esterification of RE occurred via the coupling reaction with EDC. The assignments of the other characteristic bands associated with RE and its ester derivatives are shown in Figure 2. For example, three bands at around 1600 cm^−1^, 1510 cm^−1^, and 1442 cm^−1^, attributed to the C=C stretching in the aromatic ring of RE, can be seen in both spectra. In addition, a tiny band at about 3020 cm^−1^ associated with the C–H vibration of the alkene and the aromatic ring as well as a band at 1662 cm^−1^ related to the C=C stretching of the alkene were observed. 

**Figure 3:**
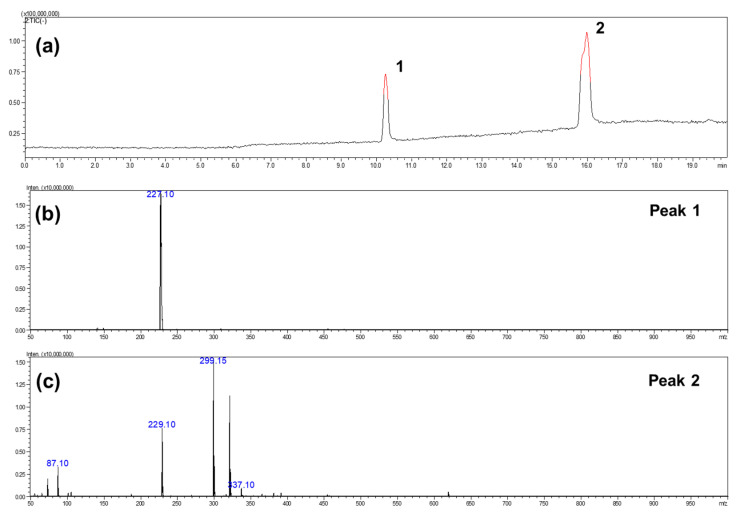
(**a**) LC chromatogram of resveratrol butyrate esters (RBE) as well as (**b**,**c**) mass spectra of peak 1 and peak 2. ^1−2^ peak of LC chromatogram.

The esterification of RE and butyric acid would likely yield three types of resveratrol ester derivatives, including monoesters, diesters, and triesters. In the present work, LC-mass spectrometry analysis was employed to identify the composition of pristine RE and RBE in the resulting product. As seen in Figure 3a, the LC chromatogram in the negative ionization mode exhibited two separated peaks. Peak 1 was detected at approximately 10.3 min, which is identical to the retention time of the pristine RE compound. The mass spectrum of peak 1, as presented in Figure 3b, had only a single signal at m/z 227.1, which was assigned to the pristine RE. These results are in agreement with those of Liu et al., who sequentially detected trans-resveratrol metabolites by LC-ESI-MS/MS [28]. The peak 2 was detected at a retention time between 15.7 min and 16.3 min and was found to exhibit several signals in its mass spectrum as shown in Figure 3c. The m/z values of resveratrol’s monoesters, diesters, and triesters were expected to be 299.1, 369.1, and 439.1, respectively. Two major signals at m/z 227.1 and 299.1, attributed to RE and its monoesters, can be found. In addition, the signals related to the diesters and triesters of resveratrol were insignificant (less than 1%). These LC-Mass spectrometry results revealed that the final product synthesized in this work was a mixture of pristine RE (approximately 26.63%) and RBE (approximately 73.37%). In addition, most of the RBE appear to be monoesters, but this result was somewhat different from the NMR analysis, therefore, it suggests RBE need further chromatographic purification in the future.

**Figure 4:**
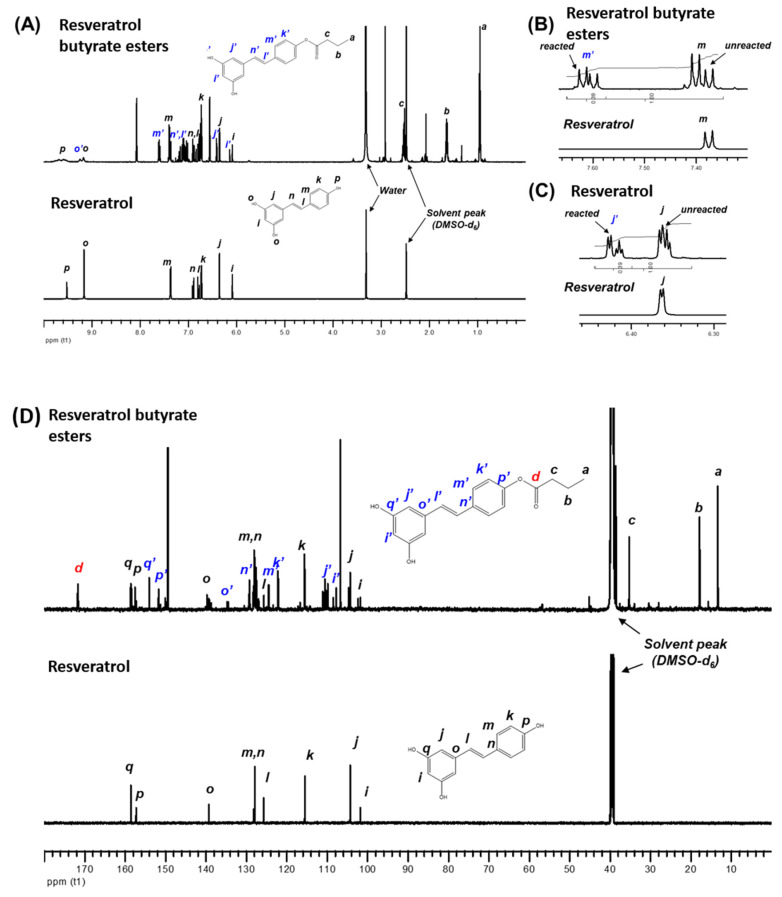
NMR spectra of resveratrol and RBE (**A**) ^1^H NMR spectra of resveratrol and RBE. (**B**) ^1^H NMR spectra of resveratrol and RBE reacted and unreacted segmentation from 7.32 to 7.66 ppm (t1). (**C**) ^1^H NMR spectra of resveratrol and RBE reacted and unreacted segmentation from 6.46 to 6.30 ppm (t1). (**D**) ^13^C NMR spectra of resveratrol and RBE.

The NMR technique was applied to confirm the chemical structure and RBE content. Figure 4 shows the ^1^H NMR spectra of RE and RBE. The NMR spectral data of RE matches that reported by Lu et al. [29], which showed that several additional signals appeared in the chemical shift range between 6 and 10 ppm after esterification. 

These signals are associated with the resveratrol ester with butyric acid. However, not all the original signals of pristine RE shifted and disappeared in the spectrum of the resveratrol ester. These findings suggested that the resulting product was a mixture, and the esterification was not complete, which is consistent with the LC-Mass spectrometry results. By comparing the integrated areas of the selected peaks, the ratio of the reacted and unreacted RE within the resulting product could be determined. 

We found that the ratio was approximately 0.38/0.62 for the signals corresponding to one of the aromatic ring protons (e.g., m and m′) at δ = 7.38 ppm and δ = 7.52 ppm. This finding indicates that the resulting product still contains 62% of pristine RE. When we considered the other signals assigned to another aromatic ring proton (e.g., j and j′) at δ = 6.36 ppm and δ = 6.42 ppm, a similar ratio of the reacted and unreacted RE (0.39/0.61) was obtained. The ^13^C NMR spectra of RE and RBE are shown in Figure 4. A signal at δ = 171.4 ppm corresponding to the carbons on the carbonyl group of the ester was found, indicating that esterification had occurred. As expected, additional peaks related to the original signals of pristine RE were detected.

Previous studies have reported that specific characteristics of the chemical structure of resveratrol determine its biological activities. It has been shown that the position of the hydroxyl groups is directly involved in its antioxidant properties, [30] and the presence of 4′–OH together with the trans stereochemistry are involved in its inhibitory effect on cell proliferation [31]. In addition, the biological properties of resveratrol are expanded through several modifications, including hydroxylation, methylation, isoprenylation, and by the formation of dimers, trimers, and oligomers [32]. Accordingly, the applications of resveratrol and its derivatives are also expanded, including the development of new anticancer agents. Oh and Shahidi [8] reported the production of twelve RE derivatives acyl chlorides of different chain lengths (C3:0–C22:6). The derivatives (RC6:0, RC8:0, RC10:0, RC12:0, RC16:0) showed better antioxidant activity in a bulk oil system. However, the resveratrol esters RC20:5n–3 (REPA) and RC22:6n–3 (RDHA) showed the highest antioxidant activity when added to ground meat. All these reports indicated that resveratrol derivatives or esterification had higher antioxidant activity in the oil system and their activities depend on the esterification position, the number of esterification substitutions, and the polymer structure. 

**Figure 5:**
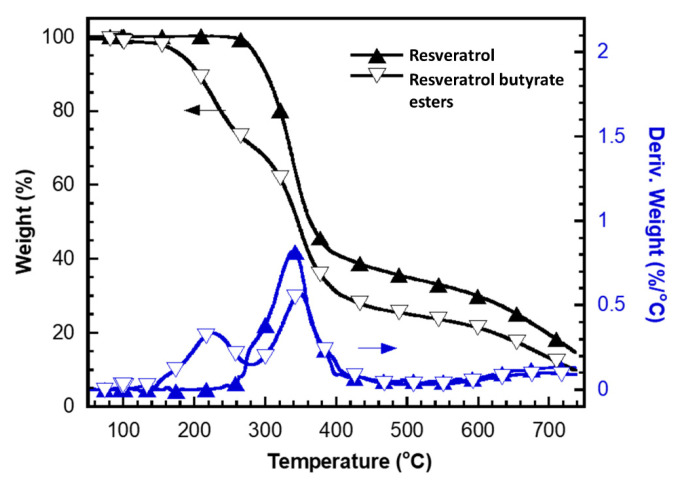
Thermograms of resveratrol and resveratrol butyrate esters under nitrogen from 50 °C to 750 °C.

The thermal gravimetric analysis (TGA) is the method of thermal analysis in which the mass of the sample is measured over time as the temperature changes, which can be used to evaluate the thermal stability of the sample, and it has been applied for investigation of the material properties in various fields such as pharmaceutical, food, and petrochemical applications [33]. TGA analysis results in Figure 5 showed that the thermal stability of RE was better than that of RBE. Both thermograms exhibited a major weight loss between 270 °C and 400 °C. This weight loss might be a result of the thermal decomposition of the phenol groups. In addition, a considerable weight loss between 140 °C and 270 °C was found only in the thermogram of RBE. This weight loss could be attributed to the degradation of the butyrate group, suggesting that the RBE was less thermally stable than RE.

**Figure 6:**
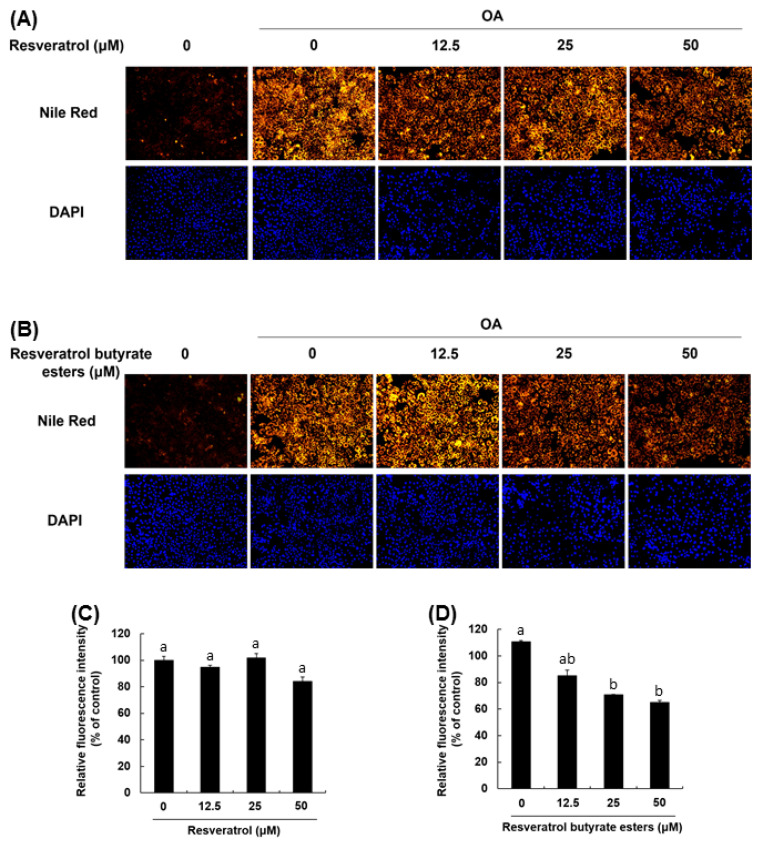
Effect of resveratrol and RBE on fat accumulation in HepG2 cells induced by treatment with 400 μM oleic acid. Nile red and DAPI stained cells treated with resveratrol (RE) (**A**) or RBE (**B**). Relative fluorescence intensity (% of control) at different RE concentrations (**C**). Relative fluorescence intensity (% of control) at different RBE concentrations (**D**). *n* = 3 per group. ^a–b^ *p* < 0.05 compared to the control.

Furthermore, we used HepG2 cells to compare the effects of RBE and RE on lipid metabolism and the underlying mechanisms. HepG2 cells were cultured with oleic acid for 48 h to induce excessive lipid accumulation. As shown in Figure 6, Nile red oil staining showed that administration of oleic acid increased lipid accumulation compared with the control group. RE, at the dose of 50 μM, reduced the levels of intracellular lipid droplets, whereas RBE showed a similar fat accumulation inhibition rate at a lower dose of 12.5 μM. At 50 μM, RBE displayed a greater inhibition on fat accumulation in a dose-dependent manner. A previous study reported that RE treatment attenuated hepatic steatosis and lowered the levels of intracellular triacylglycerides (TG) [34]. Furthermore, Western blot analysis showed that RE enhanced the phosphorylation of AMP-activated protein kinase (AMPK) and acetyl-CoA carboxylase (ACC) and downregulated the expression of sterol regulatory element-binding protein 1c (SREBP-1c) and lipin1 [34]. RE also had been shown to effectively regulate Sirt-1 and PPAR-γ and to inhibit fat accumulation [35]. Figure 6C shows that 50 μM of RE inhibited fat accumulation during Nile red staining and DAPI staining, and these results are similar to the results obtained in a previous study [36]. Here, we also found that RBE is more effective at the same concentrations, which may be associated with the change in the structure and lipophilic properties of RBE. Previous studies reported that RE derivatives also had better biological properties such as antioxidant activity and inhibition of LDL oxidation [8]. Our experiments substantiated the literature that esterification of RE could improve its biological activities.

**Figure 7:**
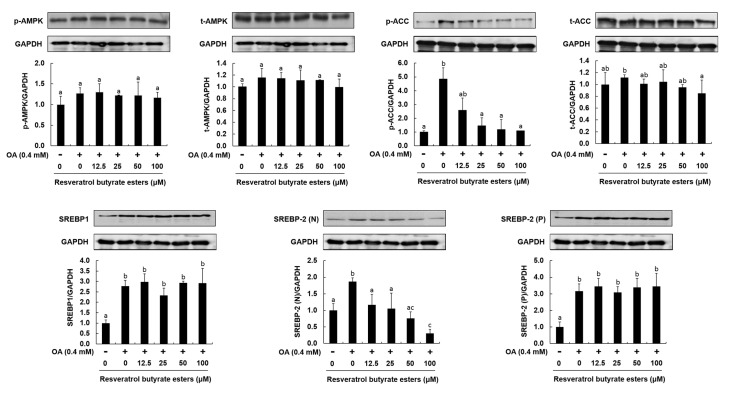
Effect of RBE on protein levels of p-AMPK, t-AMPK, p-ACC, t-ACC, SREBP-1, SREBP-2(N), and SREBP-2(p) in HepG2 cells. Histograms represent densitometric measurements of the specific bands of t-AMPK, p-ACC, t-ACC, SREBP-2(N), and SREBP-2(p) normalized for the expression levels of GAPDH, used as a control. Results are expressed as mean ± SD, *n* = 3 per group. Statistical significance was expressed as ^a–c^ *p* < 0.05 compared to the control.

RE affects host metabolism by targeting adipose tissue, skeletal muscle, liver, and intestinal microbiota [37]. These effects of RE may be partially due to the regulation of the transcription of genes involved in energy storage management or inflammation [38]. A previous study demonstrated that obese mice treated with RE had reduced levels of mRNA of genes related to the lipogenic pathway and triacylglycerol accumulation in adipose tissue [39]. Similarly, it was shown in cultured pre-adipose cells that the transcriptional effects of RE limited adipogenesis through increased mitochondrial function [40,41]. It is well known the AMPK and SREBP play an important inhibitory role in the lipogenic pathway and fat accumulation, and AMPK has emerged as a critical factor mediating the beneficial effects of polyphenols on lipid metabolic disorders [42] and energy metabolism by regulating downstream ACC and SREBP-1. Thus, these protein levels could be used as an index of cardiovascular disease and obesity [43]. The SREBP-1 targets lipogenic genes, whereas SREBP-2 is more specific to cholesterolemia gene expression, which plays an important role in controlling lipid and cholesterol metabolism [44]. Activation of SREBPs in response to a decrease in cellular sterol levels results in the acceleration of the synthesis of fatty acids, triacylglycerides, and cholesterol. Aberrant SREBP activity has been linked to metabolic diseases, such as obesity, fatty liver, insulin resistance, hyperlipidemia, and atherosclerosis. Thus, inhibition of SREBP activation might be a potential approach to mitigate metabolic disorders [45]. Numerous studies unanimously reported that the mechanism of the action of RE was associated with sirtuin 1 activation and AMPK phosphorylation. Activation of AMPK downregulates the activity of ACC by phosphorylation, resulting in inhibition of lipogenesis and increased energy metabolism [46,47]. As shown in Figure 7, RBE had no significant effects on AMPK, but could still effectively inhibit oleic acid-induced ACC phosphorylation. This finding showed that RBE could effectively increase the oxidation of fatty acids to reduce the accumulation of fatty acids. Furthermore, RBE significantly affected SREBP-1 and effectively reduced the synthesis of fatty acids (Figure 7). Therefore, this study indicates that the production through Steglich esterification of RE could enhance RE’s biological activities, and that RBE could decrease fatty acid accumulation by 34.48% through the regulation of ACC and SREBP-1. 

## 3. Materials and Methods

### 3.1. Materials

*Trans*-resveratrol was purchased from TCI Development Co., Ltd. (Shanghai, China). n-Butyric acid was procured from ACROS (Morris Plains, NJ, USA). *N*,*N*′-dicyclohexylcarbodiimide, EDAC, and 4-dimethylaminopyridine, DMAP, were supplied by Sigma-Aldrich (St. Louis, MO, USA). Dulbecco’s modified Eagle’s medium (DMEM) was supplied by Invitrogen Life Technologies (Carlsbad, MD, USA); fetal bovine serum, by Gibco-BRL (New York, NY, USA); and Bio-Rad protein assay kit, by Biorad (Hercules, CA, USA). The primary antibodies against p-AMPK, AMPK, and GAPDH were from GeneTex (Irvine, CA, USA). The primary antibodies against p-ACC, ACC, and SREPB-2 were from Cell Signaling Technology Inc. (Danvers, MA, USA).

### 3.2. Synthesis of RBE

RBE were synthesized according to a modified method by Neises and Steglich (1978) [24]. A mixture of *trans*-resveratrol (2.282 g, 10 mmol) and n-butyric acid (0.969 g, 11 mmol) was added to anhydrous tetrahydrofuran (THF) (Morris Plains, NJ, USA) (about 12 mL) in a three-neck flask with a magnetic stirrer. To perform the reaction in the dark, the flask was wrapped with aluminum foil. After all reactants were completely dissolved, predetermined amounts of EDC (1.708 g, 11 mmol) and DMPA (0.672 g, 5.5 mmol) were added into the solution. The esterification reaction was carried out by stirring the solution at room temperature under a nitrogen atmosphere for 60 h. Subsequently, the solution was poured into an excess amount of deionized water. Then, a viscous substance was precipitated. The viscous product was re-dissolved in acetone and collected, then the solvent was removed with a rotary vacuum concentrator. The concentrate of RBE was frozen at −80 °C and freeze-dried. Following freeze-drying, the light-yellow powder of RBE was obtained and stored in an opaque vial in a refrigerator at 4 °C.

### 3.3. Characterization of RE and RBE (FTIR, NMR, LC/MS, TGA)

FTIR was performed with the JASCO FTIR 460 spectrometer (Easton, MD USA). A transmission mode with 16 scans and a resolution of 2 cm^−1^ in the spectral range from 400 to 4000 cm^−1^ was applied to the analyses. In addition, we employed NMR spectroscopy to analyze the chemical structure of RE and RBE. Both ^1^H NMR and ^13^C NMR analyses were performed using the Bruker AVANCE 600 MHz NMR spectrometer, Bruker (Billerica, MA USA) with deuterated dimethyl sulfoxide (DMSO-*d*_6_) as the solvent at 30 °C. RE and RBE were analyzed using a TA Q5000, TA Instruments (New Castle, DE, USA) thermogravimetric analyzer (TGA). Their thermograms were recorded at a heating rate of 10 °C/min from 50 °C to 750 °C in a nitrogen flow (20 mL/min). The compositions of RE and RBE were determined by high-performance liquid chromatography-mass spectrometry mass spectrometry (LC/MS/MS) using an Agilent 110 HPLC unit (Agilent Technologies, Palo Alto, CA, USA). A C18 column (4.6 mm × 250 mm × 5 μm with a guard column, Sigma-Aldrich, Oakville, ON, Canada) was used for separation. The mobile phase was methanol/5% acetonitrile in water at different ratios (60:40–95:5 *v*/*v*) varying from 0 to 70 min at 0.8 mL/min, and the compounds were detected at 306 nm.

### 3.4. Cell Viability

HepG2 cells were seeded in a 96-well plate at a density of 8 × 10^3^ cells overnight. HepG2 cells were treated with RE or RBE at 1, 5, 25, 50, 75, and 100 μM for 24 h. Cell viability was determined using CellTiter-Glo^®^ One Solution Assay (Promega, Madison, WI, USA) according to the manufacturer’s instructions [48].

### 3.5. Cell Culture and Cell Treatment

HepG2 cells were cultured in DMEM (Invitrogen Life Technologies) supplemented with 10% fetal bovine serum (Gibco-BRL), 1% non-essential amino acids, and 1% antibiotic-antimycotic and incubated at 37 °C with 5% CO_2_ [48].

### 3.6. Induction of Lipid Droplet Accumulation and Staining

HepG2 cells were seeded in a 96-well plate at a density of 8 × 10^3^ cells per well and incubated overnight at 37 °C. The cells were treated with 400 μM oleic acid to stimulate lipid droplet accumulation and with vehicle or the compounds at 12.5, 25, and 50 μM for 24 h. The cells were fixed with 4% paraformaldehyde for 30 min and then stained with 1 µg/mL Nile red and 0.1 µg/mL DAPI for 15 min. The fluorescence of each sample was measured using a 550 Bio-Rad plate-reader (Bio-Rad) [49].

### 3.7. Western Blot Analysis

HepG2 cells were seeded in a 24-well plate (5 × 10^4^ cells/well) and incubated overnight at 37 °C. The cells were treated with 400 μM oleic acid and vehicle or the compounds at 12.5, 25, 50, and 100 μM for 24 h. The cells were lysed for 30 min in a radio immunoprecipitation assay (RIPA) buffer containing 1 mM phenylmethanesulfonyl fluoride. The concentration of soluble protein was determined using a DC (detergent compatible) protein assay kit (Bio-Rad). An equal amount of protein was transferred onto a polyvinylidene difluoride membrane following separation on a 12% SDS-polyacrylamide gel. Next, the membrane was blocked with 5% non-fat dry milk in Tris-buffered saline, 0.1% Tween (TBST) and shaken continuously for 2 h at room temperature. The membrane was washed once with TBST for 5 min and incubated with specific primary antibodies against p-AMPK (1:5000), AMPK (1:5000), p-ACC (1:3000), ACC (1:3000), SREPB-2 (1:2000), and GAPDH (1:8000) overnight at 4 °C. GAPDH was used as a loading control. Finally, the membrane was incubated with horseradish peroxidase-conjugated secondary antibodies (1:5000) at room temperature for 1 h. After three washes, the chemiluminescence signals were developed using an ECL (Enhanced Chemiluminescent) detection kit (PerkinElmer, Shelton, CT, USA), and the density of bands was counted using ImageJ gel analysis software [50].

### 3.8. Statistical Analyses

All experiments were conducted at least twice, and triplicate samples were used for each test. Data were collected and analyzed using one-way ANOVA and Duncan’s test. Significant differences were set at *p* < 0.05. All statistical analyses were performed using the SPSS program (version 12.0, St. Armonk, NY, USA).

## 4. Conclusions

Our study demonstrates that resveratrol butyrate esters can be produced from resveratrol and butyric acid using EDC and DMAP. FTIR, NMR, and LC/MS/MS analyses confirmed the structure of RBE, which was found to be more thermosensitive than RE. RBE reduced fat accumulation in HepG2 cells through the regulation of ACC and SREBP-1, and this effect was stronger than that of resveratrol at the same concentration. These results suggest that esterification of resveratrol improves its biological activities. The result clearly demonstrated that RBE might serve as potential anti-fat accumulation agents in functional food ingredients or additives and supplements for health promotion. Further studies are required to improve purification and confirm the effects of RBE in vivo using animal models.

## Abbreviations

RE	resveratrol
RBE	resveratrol butyrate ester
DCC	*N*,*N*′-dicyclohexylcarbodiimide
EDC	*n*-ethyl-*N*′-(3-dimethylaminopropyl) carbodiimide
DMAP	4-Dimethylaminopyridine
R.T.	room temperature
LC-ESI-MS/MS	liquid chromatography electrospray ionization tandem mass spectrometer
AMPK	AMP-activated protein kinase
SREBP	sterol regulatory element binding protein
SREBP-1c	sterol regulatory element-binding protein 1c
